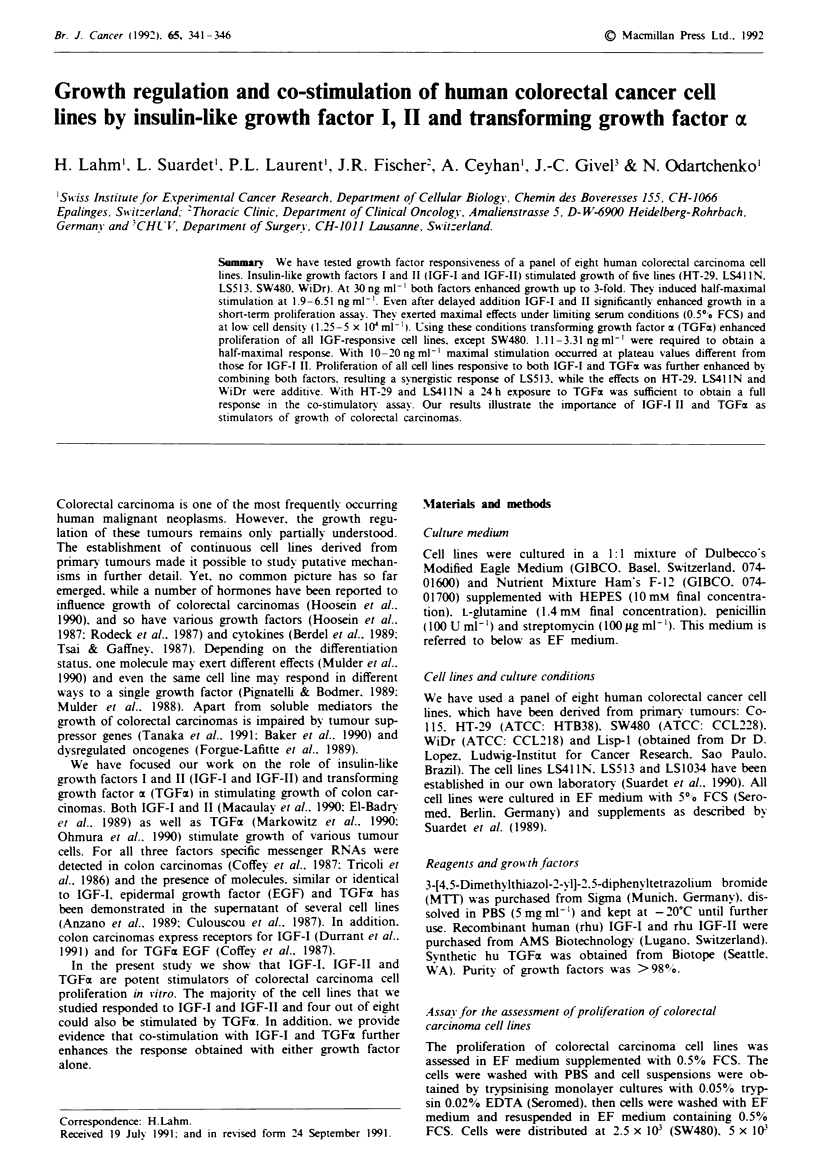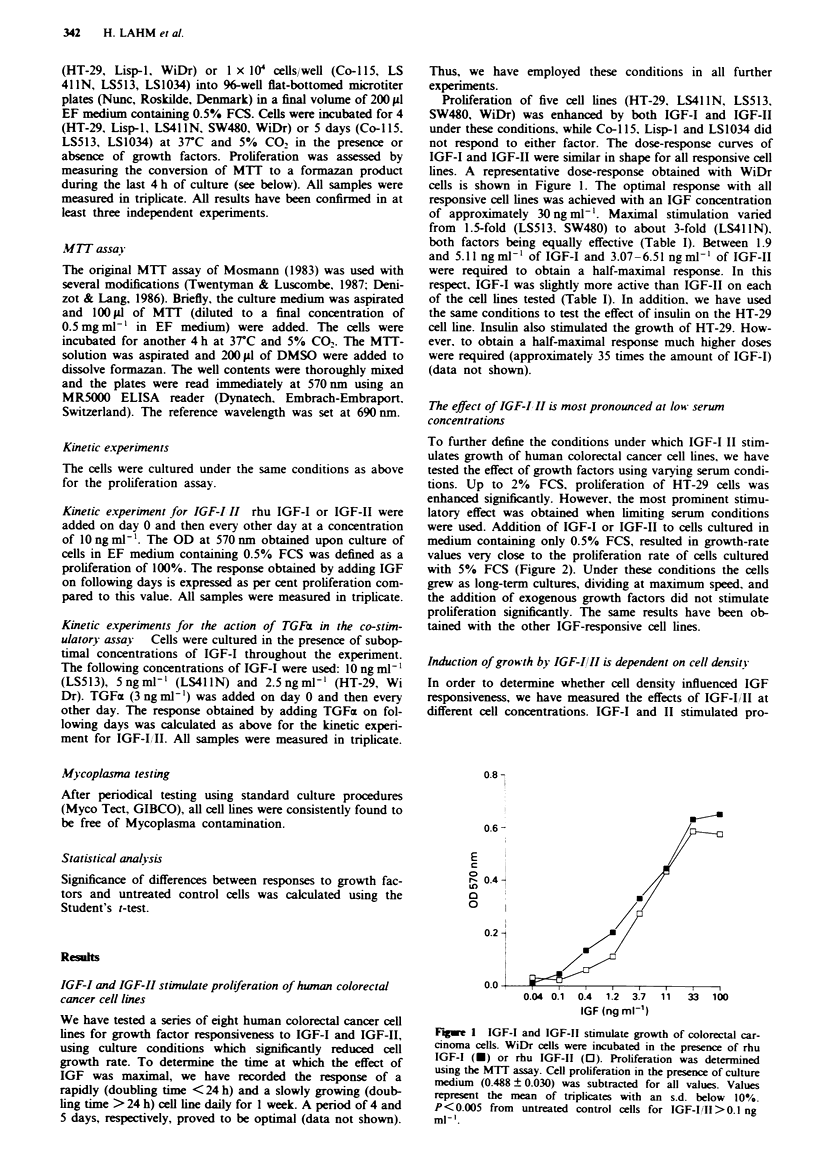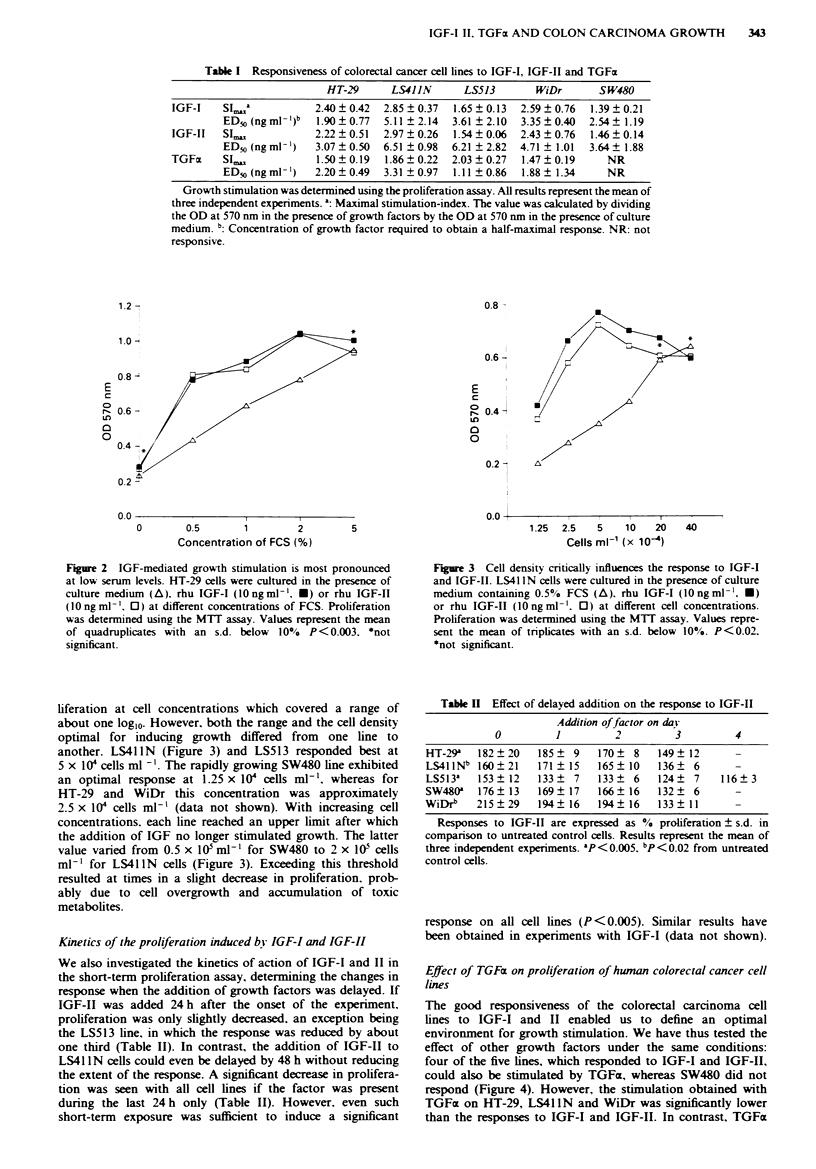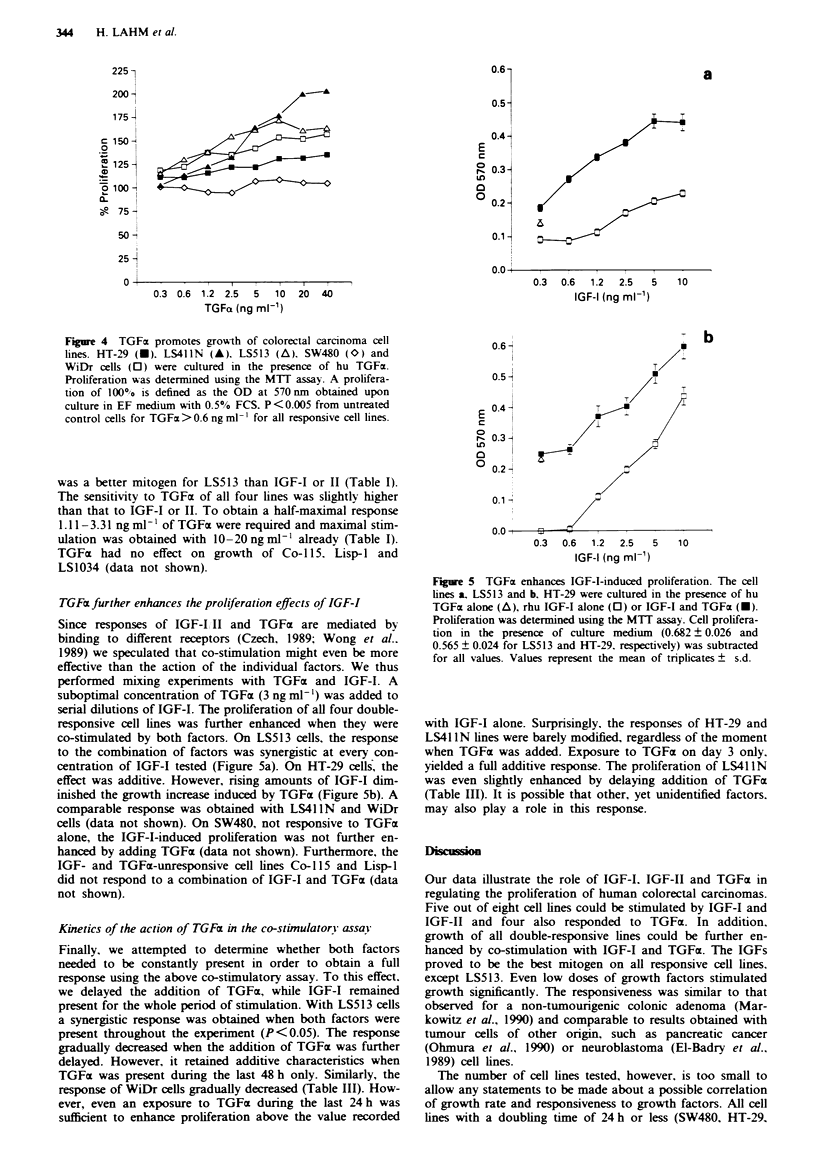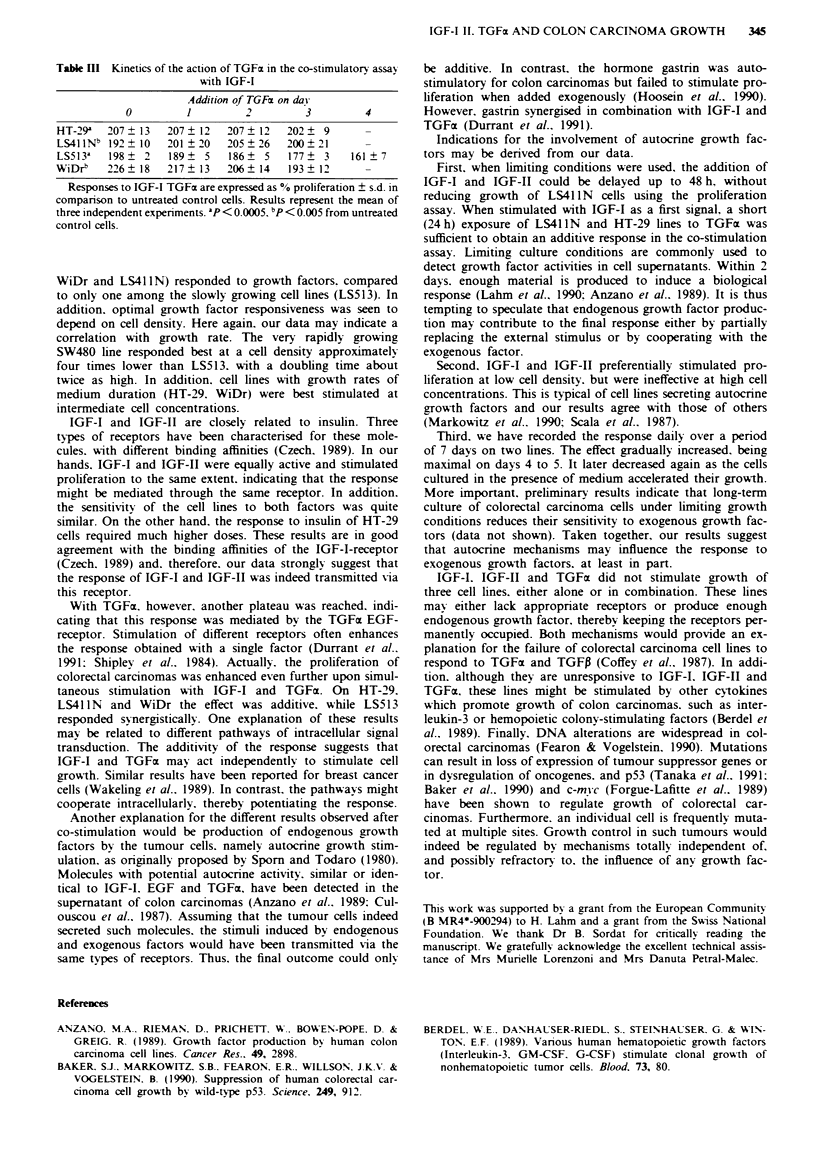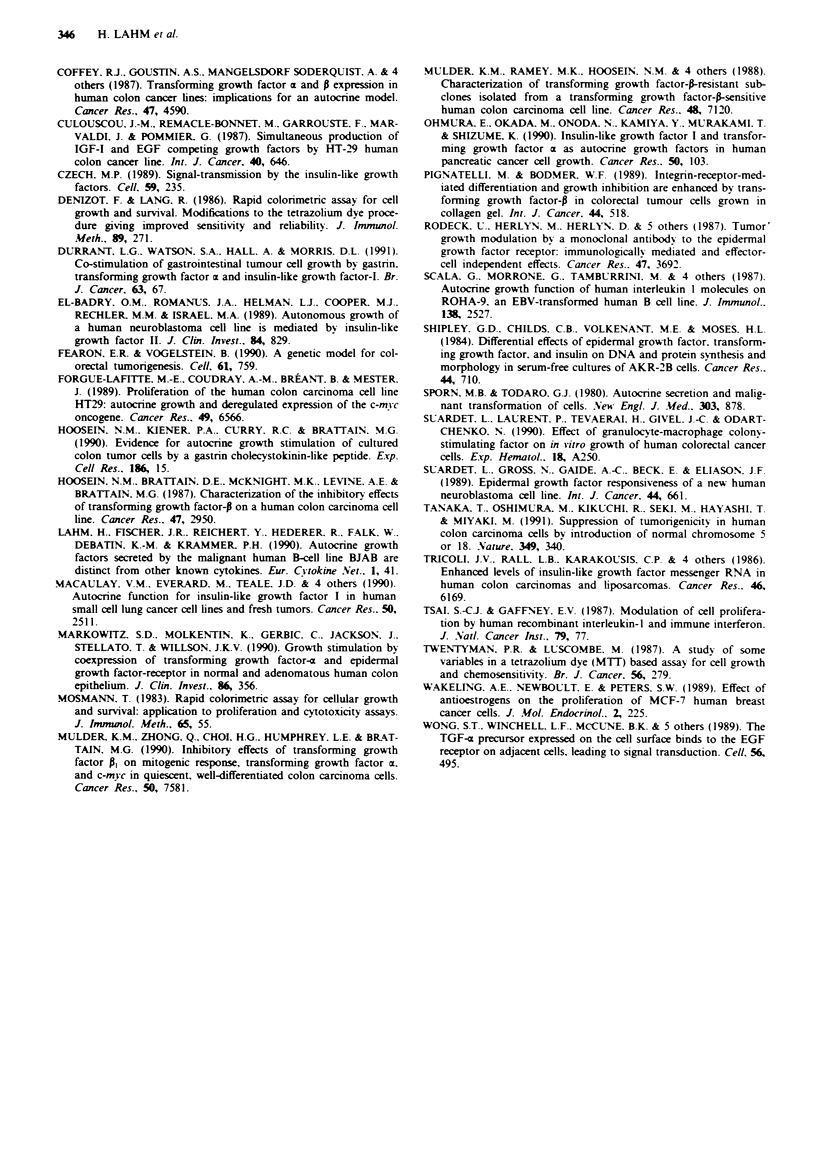# Growth regulation and co-stimulation of human colorectal cancer cell lines by insulin-like growth factor I, II and transforming growth factor alpha.

**DOI:** 10.1038/bjc.1992.69

**Published:** 1992-03

**Authors:** H. Lahm, L. Suardet, P. L. Laurent, J. R. Fischer, A. Ceyhan, J. C. Givel, N. Odartchenko

**Affiliations:** Swiss Institute for Experimental Cancer Research, Department of Cellular Biology, Epalinges.

## Abstract

We have tested growth factor responsiveness of a panel of eight human colorectal carcinoma cell lines. Insulin-like growth factors I and II (IGF-I and IGF-II) stimulated growth of five lines (HT-29, LS411N, LS513, SW480, WiDr). At 30 ng ml-1 both factors enhanced growth up to 3-fold. They induced half-maximal stimulation at 1.9-6.51 ng ml-1. Even after delayed addition IGF-I and II significantly enhanced growth in a short-term proliferation assay. They exerted maximal effects under limiting serum conditions (0.5% FCS) and at low cell density (1.25-5 x 10(4) ml-1). Using these conditions transforming growth factor alpha (TGF alpha) enhanced proliferation of all IGF-responsive cell lines, except SW480. 1.11-3.31 ng ml-1 were required to obtain a half-maximal response. With 10-20 ng ml-1 maximal stimulation occurred at plateau values different from those for IGF-I/II. Proliferation of all cell lines responsive to both IGF-I and TGF alpha was further enhanced by combining both factors, resulting a synergistic response of LS513, while the effects on HT-29, LS411N and WiDr were additive. With HT-29 and LS411N a 24 h exposure to TGF alpha was sufficient to obtain a full response in the co-stimulatory assay. Our results illustrate the importance of IGF-I/II and TGF alpha as stimulators of growth of colorectal carcinomas.


Br. J. Cancer (1992), 65, 341-346 © Macmillan Press Ltd., 1992

### Growth regulation and co-stimulation of human colorectal cancer cell lines by insulin-like growth factor I, II and transforming growth factor α

H. Lahm[1], L. Suardet[1], P.L. Laurent[1], J.R. Fischer[2], A. Ceyhan[1], J.-C. Givel[3] & N. Odartchenko[1]

[1]*Swiss Institute for Experimental Cancer Research, Department of Cellular Biology, Chemin des Boveresses 155, CH-1066 Epalinges, Switzerland;* [2]*Thoracic Clinic, Department of Clinical Oncology, Amalienstrasse 5, D-W-6900 Heidelberg-Rohrbach, Germany and* [3]*CHUV, Department of Surgery, CH-1011 Lausanne, Switzerland.*

**Summary** We have tested growth factor responsiveness of a panel of eight human colorectal carcinoma cell lines. Insulin-like growth factors I and II (IGF-I and IGF-II) stimulated growth of five lines (HT-29, LS411N, LS513, SW480, WiDr). At 30 ng ml$^{-1}$ both factors enhanced growth up to 3-fold. They induced half-maximal stimulation at 1.9-6.51 ng ml$^{-1}$. Even after delayed addition IGF-I and II significantly enhanced growth in a short-term proliferation assay. They exerted maximal effects under limiting serum conditions (0.5% FCS) and at low cell density (1.25-5 × 10$^4$ ml$^{-1}$). Using these conditions transforming growth factor α (TGFα) enhanced proliferation of all IGF-responsive cell lines, except SW480. 1.11-3.31 ng ml$^{-1}$ were required to obtain a half-maximal response. With 10-20 ng ml$^{-1}$ maximal stimulation occurred at plateau values different from those for IGF-I II. Proliferation of all cell lines responsive to both IGF-I and TGFα was further enhanced by combining both factors, resulting a synergistic response of LS513, while the effects on HT-29, LS411N and WiDr were additive. With HT-29 and LS411N a 24 h exposure to TGFα was sufficient to obtain a full response in the co-stimulatory assay. Our results illustrate the importance of IGF-I II and TGFα as stimulators of growth of colorectal carcinomas.

Colorectal carcinoma is one of the most frequently occurring human malignant neoplasms. However, the growth regulation of these tumours remains only partially understood. The establishment of continuous cell lines derived from primary tumours made it possible to study putative mechanisms in further detail. Yet, no common picture has so far emerged, while a number of hormones have been reported to influence growth of colorectal carcinomas (Hoosein *et al.*, 1990), and so have various growth factors (Hoosein *et al.*, 1987; Rodeck *et al.*, 1987) and cytokines (Berdel *et al.*, 1989; Tsai & Gaffney, 1987). Depending on the differentiation status, one molecule may exert different effects (Mulder *et al.*, 1990) and even the same cell line may respond in different ways to a single growth factor (Pignatelli & Bodmer, 1989; Mulder *et al.*, 1988). Apart from soluble mediators the growth of colorectal carcinomas is impaired by tumour suppressor genes (Tanaka *et al.*, 1991; Baker *et al.*, 1990) and dysregulated oncogenes (Forgue-Lafitte *et al.*, 1989).

We have focused our work on the role of insulin-like growth factors I and II (IGF-I and IGF-II) and transforming growth factor α (TGFα) in stimulating growth of colon carcinomas. Both IGF-I and II (Macaulay *et al.*, 1990; El-Badry *et al.*, 1989) as well as TGFα (Markowitz *et al.*, 1990; Ohmura *et al.*, 1990) stimulate growth of various tumour cells. For all three factors specific messenger RNAs were detected in colon carcinomas (Coffey *et al.*, 1987; Tricoli *et al.*, 1986) and the presence of molecules, similar or identical to IGF-I, epidermal growth factor (EGF) and TGFα has been demonstrated in the supernatant of several cell lines (Anzano *et al.*, 1989; Culouscou *et al.*, 1987). In addition, colon carcinomas express receptors for IGF-I (Durrant *et al.*, 1991) and for TGFα EGF (Coffey *et al.*, 1987).

In the present study we show that IGF-I, IGF-II and TGFα are potent stimulators of colorectal carcinoma cell proliferation *in vitro*. The majority of the cell lines that we studied responded to IGF-I and IGF-II and four out of eight could also be stimulated by TGFα. In addition, we provide evidence that co-stimulation with IGF-I and TGFα further enhances the response obtained with either growth factor alone.

Correspondence: H.Lahm.

Received 19 July 1991; and in revised form 24 September 1991.

### Materials and methods

### Culture medium

Cell lines were cultured in a 1:1 mixture of Dulbecco's Modified Eagle Medium (GIBCO, Basel, Switzerland, 074-01600) and Nutrient Mixture Ham's F-12 (GIBCO, 074-01700) supplemented with HEPES (10 mM final concentration), L-glutamine (1.4 mM final concentration), penicillin (100 U ml$^{-1}$) and streptomycin (100 μg ml$^{-1}$). This medium is referred to below as EF medium.

### Cell lines and culture conditions

We have used a panel of eight human colorectal cancer cell lines, which have been derived from primary tumours: Co-115, HT-29 (ATCC: HTB38), SW480 (ATCC: CCL228), WiDr (ATCC: CCL218) and Lisp-1 (obtained from Dr D. Lopez, Ludwig-Institut for Cancer Research, Sao Paulo, Brazil). The cell lines LS411N, LS513 and LS1034 have been established in our own laboratory (Suardet *et al.*, 1990). All cell lines were cultured in EF medium with 5% FCS (Seromed, Berlin, Germany) and supplements as described by Suardet *et al.* (1989).

### Reagents and growth factors

3-[4,5-Dimethylthiazol-2-yl]-2,5-diphenyltetrazolium bromide (MTT) was purchased from Sigma (Munich, Germany), dissolved in PBS (5 mg ml$^{-1}$) and kept at −20°C until further use. Recombinant human (rhu) IGF-I and rhu IGF-II were purchased from AMS Biotechnology (Lugano, Switzerland). Synthetic hu TGFα was obtained from Biotope (Seattle, WA). Purity of growth factors was >98%.

### Assay for the assessment of proliferation of colorectal carcinoma cell lines

The proliferation of colorectal carcinoma cell lines was assessed in EF medium supplemented with 0.5% FCS. The cells were washed with PBS and cell suspensions were obtained by trypsinising monolayer cultures with 0.05% trypsin 0.02% EDTA (Seromed), then cells were washed with EF medium and resuspended in EF medium containing 0.5% FCS. Cells were distributed at 2.5 × 10$^3$ (SW480), 5 × 10$^3$

(HT-29, Lisp-1, WiDr) or $1 \times 10^4$ cells/well (Co-115, LS 411N, LS513, LS1034) into 96-well flat-bottomed microtiter plates (Nunc, Roskilde, Denmark) in a final volume of 200 µl EF medium containing 0.5% FCS. Cells were incubated for 4 (HT-29, Lisp-1, LS411N, SW480, WiDr) or 5 days (Co-115, LS513, LS1034) at 37°C and 5% $CO_2$ in the presence or absence of growth factors. Proliferation was assessed by measuring the conversion of MTT to a formazan product during the last 4 h of culture (see below). All samples were measured in triplicate. All results have been confirmed in at least three independent experiments.

*MTT assay*

The original MTT assay of Mosmann (1983) was used with several modifications (Twentyman & Luscombe, 1987; Denizot & Lang, 1986). Briefly, the culture medium was aspirated and 100 µl of MTT (diluted to a final concentration of 0.5 mg $ml^{-1}$ in EF medium) were added. The cells were incubated for another 4 h at 37°C and 5% $CO_2$. The MTT-solution was aspirated and 200 µl of DMSO were added to dissolve formazan. The well contents were thoroughly mixed and the plates were read immediately at 570 nm using an MR5000 ELISA reader (Dynatech, Embrach-Embraport, Switzerland). The reference wavelength was set at 690 nm.

*Kinetic experiments*

The cells were cultured under the same conditions as above for the proliferation assay.

*Kinetic experiment for IGF-I/II* rhu IGF-I or IGF-II were added on day 0 and then every other day at a concentration of 10 ng $ml^{-1}$. The OD at 570 nm obtained upon culture of cells in EF medium containing 0.5% FCS was defined as a proliferation of 100%. The response obtained by adding IGF on following days is expressed as per cent proliferation compared to this value. All samples were measured in triplicate.

*Kinetic experiments for the action of TGFα in the co-stimulatory assay* Cells were cultured in the presence of suboptimal concentrations of IGF-I throughout the experiment. The following concentrations of IGF-I were used: 10 ng $ml^{-1}$ (LS513), 5 ng $ml^{-1}$ (LS411N) and 2.5 ng $ml^{-1}$ (HT-29, Wi Dr). TGFα (3 ng $ml^{-1}$) was added on day 0 and then every other day. The response obtained by adding TGFα on following days was calculated as above for the kinetic experiment for IGF-I/II. All samples were measured in triplicate.

*Mycoplasma testing*

After periodical testing using standard culture procedures (Myco Tect, GIBCO), all cell lines were consistently found to be free of Mycoplasma contamination.

*Statistical analysis*

Significance of differences between responses to growth factors and untreated control cells was calculated using the Student's $t$-test.

### Results

*IGF-I and IGF-II stimulate proliferation of human colorectal cancer cell lines*

We have tested a series of eight human colorectal cancer cell lines for growth factor responsiveness to IGF-I and IGF-II, using culture conditions which significantly reduced cell growth rate. To determine the time at which the effect of IGF was maximal, we have recorded the response of a rapidly (doubling time <24 h) and a slowly growing (doubling time >24 h) cell line daily for 1 week. A period of 4 and 5 days, respectively, proved to be optimal (data not shown). Thus, we have employed these conditions in all further experiments.

Proliferation of five cell lines (HT-29, LS411N, LS513, SW480, WiDr) was enhanced by both IGF-I and IGF-II under these conditions, while Co-115, Lisp-1 and LS1034 did not respond to either factor. The dose-response curves of IGF-I and IGF-II were similar in shape for all responsive cell lines. A representative dose-response obtained with WiDr cells is shown in Figure 1. The optimal response with all responsive cell lines was achieved with an IGF concentration of approximately 30 ng $ml^{-1}$. Maximal stimulation varied from 1.5-fold (LS513, SW480) to about 3-fold (LS411N), both factors being equally effective (Table I). Between 1.9 and 5.11 ng $ml^{-1}$ of IGF-I and 3.07–6.51 ng $ml^{-1}$ of IGF-II were required to obtain a half-maximal response. In this respect, IGF-I was slightly more active than IGF-II on each of the cell lines tested (Table I). In addition, we have used the same conditions to test the effect of insulin on the HT-29 cell line. Insulin also stimulated the growth of HT-29. However, to obtain a half-maximal response much higher doses were required (approximately 35 times the amount of IGF-I) (data not shown).

*The effect of IGF-I/II is most pronounced at low serum concentrations*

To further define the conditions under which IGF-I/II stimulates growth of human colorectal cancer cell lines, we have tested the effect of growth factors using varying serum conditions. Up to 2% FCS, proliferation of HT-29 cells was enhanced significantly. However, the most prominent stimulatory effect was obtained when limiting serum conditions were used. Addition of IGF-I or IGF-II to cells cultured in medium containing only 0.5% FCS, resulted in growth-rate values very close to the proliferation rate of cells cultured with 5% FCS (Figure 2). Under these conditions the cells grew as long-term cultures, dividing at maximum speed, and the addition of exogenous growth factors did not stimulate proliferation significantly. The same results have been obtained with the other IGF-responsive cell lines.

*Induction of growth by IGF-I/II is dependent on cell density*

In order to determine whether cell density influenced IGF responsiveness, we have measured the effects of IGF-I/II at different cell concentrations. IGF-I and II stimulated pro-

**Figure 1** IGF-I and IGF-II stimulate growth of colorectal carcinoma cells. WiDr cells were incubated in the presence of rhu IGF-I (■) or rhu IGF-II (□). Proliferation was determined using the MTT assay. Cell proliferation in the presence of culture medium (0.488 ± 0.030) was subtracted for all values. Values represent the mean of triplicates with an s.d. below 10%. $P<0.005$ from untreated control cells for IGF-I/II>0.1 ng $ml^{-1}$.

**Table I** Responsiveness of colorectal cancer cell lines to IGF-I, IGF-II and TGFα

| | | *HT-29* | *LS411N* | *LS513* | *WiDr* | *SW480* |
|---|---|---|---|---|---|---|
| IGF-I | $SI_{max}$[a] | 2.40 ± 0.42 | 2.85 ± 0.37 | 1.65 ± 0.13 | 2.59 ± 0.76 | 1.39 ± 0.21 |
| | $ED_{50}$ (ng ml$^{-1}$)[b] | 1.90 ± 0.77 | 5.11 ± 2.14 | 3.61 ± 2.10 | 3.35 ± 0.40 | 2.54 ± 1.19 |
| IGF-II | $SI_{max}$ | 2.22 ± 0.51 | 2.97 ± 0.26 | 1.54 ± 0.06 | 2.43 ± 0.76 | 1.46 ± 0.14 |
| | $ED_{50}$ (ng ml$^{-1}$) | 3.07 ± 0.50 | 6.51 ± 0.98 | 6.21 ± 2.82 | 4.71 ± 1.01 | 3.64 ± 1.88 |
| TGFα | $SI_{max}$ | 1.50 ± 0.19 | 1.86 ± 0.22 | 2.03 ± 0.27 | 1.47 ± 0.19 | NR |
| | $ED_{50}$ (ng ml$^{-1}$) | 2.20 ± 0.49 | 3.31 ± 0.97 | 1.11 ± 0.86 | 1.88 ± 1.34 | NR |

Growth stimulation was determined using the proliferation assay. All results represent the mean of three independent experiments. [a]: Maximal stimulation-index. The value was calculated by dividing the OD at 570 nm in the presence of growth factors by the OD at 570 nm in the presence of culture medium. [b]: Concentration of growth factor required to obtain a half-maximal response. NR: not responsive.

**Figure 2** IGF-mediated growth stimulation is most pronounced at low serum levels. HT-29 cells were cultured in the presence of culture medium (△), rhu IGF-I (10 ng ml$^{-1}$, ■) or rhu IGF-II (10 ng ml$^{-1}$, □) at different concentrations of FCS. Proliferation was determined using the MTT assay. Values represent the mean of quadruplicates with an s.d. below 10% $P<0.003$. *not significant.

**Figure 3** Cell density critically influences the response to IGF-I and IGF-II. LS411N cells were cultured in the presence of culture medium containing 0.5% FCS (△), rhu IGF-I (10 ng ml$^{-1}$, ■) or rhu IGF-II (10 ng ml$^{-1}$, □) at different cell concentrations. Proliferation was determined using the MTT assay. Values represent the mean of triplicates with an s.d. below 10%. $P<0.02$. *not significant.

liferation at cell concentrations which covered a range of about one $\log_{10}$. However, both the range and the cell density optimal for inducing growth differed from one line to another. LS411N (Figure 3) and LS513 responded best at 5 × 10$^4$ cells ml$^{-1}$. The rapidly growing SW480 line exhibited an optimal response at 1.25 × 10$^4$ cells ml$^{-1}$, whereas for HT-29 and WiDr this concentration was approximately 2.5 × 10$^4$ cells ml$^{-1}$ (data not shown). With increasing cell concentrations, each line reached an upper limit after which the addition of IGF no longer stimulated growth. The latter value varied from 0.5 × 10$^5$ ml$^{-1}$ for SW480 to 2 × 10$^5$ cells ml$^{-1}$ for LS411N cells (Figure 3). Exceeding this threshold resulted at times in a slight decrease in proliferation, probably due to cell overgrowth and accumulation of toxic metabolites.

### *Kinetics of the proliferation induced by IGF-I and IGF-II*

We also investigated the kinetics of action of IGF-I and II in the short-term proliferation assay, determining the changes in response when the addition of growth factors was delayed. If IGF-II was added 24 h after the onset of the experiment, proliferation was only slightly decreased, an exception being the LS513 line, in which the response was reduced by about one third (Table II). In contrast, the addition of IGF-II to LS411N cells could even be delayed by 48 h without reducing the extent of the response. A significant decrease in proliferation was seen with all cell lines if the factor was present during the last 24 h only (Table II). However, even such short-term exposure was sufficient to induce a significant response on all cell lines ($P<0.005$). Similar results have been obtained in experiments with IGF-I (data not shown).

**Table II** Effect of delayed addition on the response to IGF-II

| | *Addition of factor on day* | | | | |
|---|---|---|---|---|---|
| | *0* | *1* | *2* | *3* | *4* |
| HT-29[a] | 182 ± 20 | 185 ± 9 | 170 ± 8 | 149 ± 12 | – |
| LS411N[b] | 160 ± 21 | 171 ± 15 | 165 ± 10 | 136 ± 6 | – |
| LS513[a] | 153 ± 12 | 133 ± 7 | 133 ± 6 | 124 ± 7 | 116 ± 3 |
| SW480[a] | 176 ± 13 | 169 ± 17 | 166 ± 16 | 132 ± 6 | – |
| WiDr[b] | 215 ± 29 | 194 ± 16 | 194 ± 16 | 133 ± 11 | – |

Responses to IGF-II are expressed as % proliferation ± s.d. in comparison to untreated control cells. Results represent the mean of three independent experiments. [a]$P<0.005$, [b]$P<0.02$ from untreated control cells.

### *Effect of TGFα on proliferation of human colorectal cancer cell lines*

The good responsiveness of the colorectal carcinoma cell lines to IGF-I and II enabled us to define an optimal environment for growth stimulation. We have thus tested the effect of other growth factors under the same conditions: four of the five lines, which responded to IGF-I and IGF-II, could also be stimulated by TGFα, whereas SW480 did not respond (Figure 4). However, the stimulation obtained with TGFα on HT-29, LS411N and WiDr was significantly lower than the responses to IGF-I and IGF-II. In contrast, TGFα

**Figure 4** TGFα promotes growth of colorectal carcinoma cell lines. HT-29 (■), LS411N (▲), LS513 (△), SW480 (◇) and WiDr cells (□) were cultured in the presence of hu TGFα. Proliferation was determined using the MTT assay. A proliferation of 100% is defined as the OD at 570 nm obtained upon culture in EF medium with 0.5% FCS. $P < 0.005$ from untreated control cells for TGFα > 0.6 ng $ml^{-1}$ for all responsive cell lines.

**Figure 5** TGFα enhances IGF-I-induced proliferation. The cell lines **a**, LS513 and **b**, HT-29 were cultured in the presence of hu TGFα alone (△), rhu IGF-I alone (□) or IGF-I and TGFα (■). Proliferation was determined using the MTT assay. Cell proliferation in the presence of culture medium (0.682 ± 0.026 and 0.565 ± 0.024 for LS513 and HT-29, respectively) was subtracted for all values. Values represent the mean of triplicates ± s.d.

was a better mitogen for LS513 than IGF-I or II (Table I). The sensitivity to TGFα of all four lines was slightly higher than that to IGF-I or II. To obtain a half-maximal response 1.11–3.31 ng $ml^{-1}$ of TGFα were required and maximal stimulation was obtained with 10–20 ng $ml^{-1}$ already (Table I). TGFα had no effect on growth of Co-115, Lisp-1 and LS1034 (data not shown).

*TGFα further enhances the proliferation effects of IGF-I*

Since responses of IGF-I/II and TGFα are mediated by binding to different receptors (Czech, 1989; Wong *et al.*, 1989) we speculated that co-stimulation might even be more effective than the action of the individual factors. We thus performed mixing experiments with TGFα and IGF-I. A suboptimal concentration of TGFα (3 ng $ml^{-1}$) was added to serial dilutions of IGF-I. The proliferation of all four double-responsive cell lines was further enhanced when they were co-stimulated by both factors. On LS513 cells, the response to the combination of factors was synergistic at every concentration of IGF-I tested (Figure 5a). On HT-29 cells, the effect was additive. However, rising amounts of IGF-I diminished the growth increase induced by TGFα (Figure 5b). A comparable response was obtained with LS411N and WiDr cells (data not shown). On SW480, not responsive to TGFα alone, the IGF-I-induced proliferation was not further enhanced by adding TGFα (data not shown). Furthermore, the IGF- and TGFα-unresponsive cell lines Co-115 and Lisp-1 did not respond to a combination of IGF-I and TGFα (data not shown).

*Kinetics of the action of TGFα in the co-stimulatory assay*

Finally, we attempted to determine whether both factors needed to be constantly present in order to obtain a full response using the above co-stimulatory assay. To this effect, we delayed the addition of TGFα, while IGF-I remained present for the whole period of stimulation. With LS513 cells a synergistic response was obtained when both factors were present throughout the experiment ($P < 0.05$). The response gradually decreased when the addition of TGFα was further delayed. However, it retained additive characteristics when TGFα was present during the last 48 h only. Similarly, the response of WiDr cells gradually decreased (Table III). However, even an exposure to TGFα during the last 24 h was sufficient to enhance proliferation above the value recorded with IGF-I alone. Surprisingly, the responses of HT-29 and LS411N lines were barely modified, regardless of the moment when TGFα was added. Exposure to TGFα on day 3 only, yielded a full additive response. The proliferation of LS411N was even slightly enhanced by delaying addition of TGFα (Table III). It is possible that other, yet unidentified factors, may also play a role in this response.

### Discussion

Our data illustrate the role of IGF-I, IGF-II and TGFα in regulating the proliferation of human colorectal carcinomas. Five out of eight cell lines could be stimulated by IGF-I and IGF-II and four also responded to TGFα. In addition, growth of all double-responsive lines could be further enhanced by co-stimulation with IGF-I and TGFα. The IGFs proved to be the best mitogen on all responsive cell lines, except LS513. Even low doses of growth factors stimulated growth significantly. The responsiveness was similar to that observed for a non-tumourigenic colonic adenoma (Markowitz *et al.*, 1990) and comparable to results obtained with tumour cells of other origin, such as pancreatic cancer (Ohmura *et al.*, 1990) or neuroblastoma (El-Badry *et al.*, 1989) cell lines.

The number of cell lines tested, however, is too small to allow any statements to be made about a possible correlation of growth rate and responsiveness to growth factors. All cell lines with a doubling time of 24 h or less (SW480, HT-29,

**Table III** Kinetics of the action of TGFα in the co-stimulatory assay with IGF-I

| | *Addition of TGFα on day* | | | | |
|---|---|---|---|---|---|
| | *0* | *1* | *2* | *3* | *4* |
| HT-29[a] | 207 ± 13 | 207 ± 12 | 207 ± 12 | 202 ± 9 | – |
| LS411N[b] | 192 ± 10 | 201 ± 20 | 205 ± 26 | 200 ± 21 | – |
| LS513[a] | 198 ± 2 | 189 ± 5 | 186 ± 5 | 177 ± 3 | 161 ± 7 |
| WiDr[b] | 226 ± 18 | 217 ± 13 | 206 ± 14 | 193 ± 12 | – |

Responses to IGF-I TGFα are expressed as % proliferation ± s.d. in comparison to untreated control cells. Results represent the mean of three independent experiments. [a]$P < 0.0005$, [b]$P < 0.005$ from untreated control cells.

WiDr and LS411N) responded to growth factors, compared to only one among the slowly growing cell lines (LS513). In addition, optimal growth factor responsiveness was seen to depend on cell density. Here again, our data may indicate a correlation with growth rate. The very rapidly growing SW480 line responded best at a cell density approximately four times lower than LS513, with a doubling time about twice as high. In addition, cell lines with growth rates of medium duration (HT-29, WiDr) were best stimulated at intermediate cell concentrations.

IGF-I and IGF-II are closely related to insulin. Three types of receptors have been characterised for these molecules, with different binding affinities (Czech, 1989). In our hands, IGF-I and IGF-II were equally active and stimulated proliferation to the same extent, indicating that the response might be mediated through the same receptor. In addition, the sensitivity of the cell lines to both factors was quite similar. On the other hand, the response to insulin of HT-29 cells required much higher doses. These results are in good agreement with the binding affinities of the IGF-I-receptor (Czech, 1989) and, therefore, our data strongly suggest that the response of IGF-I and IGF-II was indeed transmitted via this receptor.

With TGFα, however, another plateau was reached, indicating that this response was mediated by the TGFα EGF-receptor. Stimulation of different receptors often enhances the response obtained with a single factor (Durrant *et al.*, 1991; Shipley *et al.*, 1984). Actually, the proliferation of colorectal carcinomas was enhanced even further upon simultaneous stimulation with IGF-I and TGFα. On HT-29, LS411N and WiDr the effect was additive, while LS513 responded synergistically. One explanation of these results may be related to different pathways of intracellular signal transduction. The additivity of the response suggests that IGF-I and TGFα may act independently to stimulate cell growth. Similar results have been reported for breast cancer cells (Wakeling *et al.*, 1989). In contrast, the pathways might cooperate intracellularly, thereby potentiating the response.

Another explanation for the different results observed after co-stimulation would be production of endogenous growth factors by the tumour cells, namely autocrine growth stimulation, as originally proposed by Sporn and Todaro (1980). Molecules with potential autocrine activity, similar or identical to IGF-I, EGF and TGFα, have been detected in the supernatant of colon carcinomas (Anzano *et al.*, 1989; Culouscou *et al.*, 1987). Assuming that the tumour cells indeed secreted such molecules, the stimuli induced by endogenous and exogenous factors would have been transmitted via the same types of receptors. Thus, the final outcome could only be additive. In contrast, the hormone gastrin was autostimulatory for colon carcinomas but failed to stimulate proliferation when added exogenously (Hoosein *et al.*, 1990). However, gastrin synergised in combination with IGF-I and TGFα (Durrant *et al.*, 1991).

Indications for the involvement of autocrine growth factors may be derived from our data.

First, when limiting conditions were used, the addition of IGF-I and IGF-II could be delayed up to 48 h, without reducing growth of LS411N cells using the proliferation assay. When stimulated with IGF-I as a first signal, a short (24 h) exposure of LS411N and HT-29 lines to TGFα was sufficient to obtain an additive response in the co-stimulation assay. Limiting culture conditions are commonly used to detect growth factor activities in cell supernatants. Within 2 days, enough material is produced to induce a biological response (Lahm *et al.*, 1990; Anzano *et al.*, 1989). It is thus tempting to speculate that endogenous growth factor production may contribute to the final response either by partially replacing the external stimulus or by cooperating with the exogenous factor.

Second, IGF-I and IGF-II preferentially stimulated proliferation at low cell density, but were ineffective at high cell concentrations. This is typical of cell lines secreting autocrine growth factors and our results agree with those of others (Markowitz *et al.*, 1990; Scala *et al.*, 1987).

Third, we have recorded the response daily over a period of 7 days on two lines. The effect gradually increased, being maximal on days 4 to 5. It later decreased again as the cells cultured in the presence of medium accelerated their growth. More important, preliminary results indicate that long-term culture of colorectal carcinoma cells under limiting growth conditions reduces their sensitivity to exogenous growth factors (data not shown). Taken together, our results suggest that autocrine mechanisms may influence the response to exogenous growth factors, at least in part.

IGF-I, IGF-II and TGFα did not stimulate growth of three cell lines, either alone or in combination. These lines may either lack appropriate receptors or produce enough endogenous growth factor, thereby keeping the receptors permanently occupied. Both mechanisms would provide an explanation for the failure of colorectal carcinoma cell lines to respond to TGFα and TGFβ (Coffey *et al.*, 1987). In addition, although they are unresponsive to IGF-I, IGF-II and TGFα, these lines might be stimulated by other cytokines which promote growth of colon carcinomas, such as interleukin-3 or hemopoietic colony-stimulating factors (Berdel *et al.*, 1989). Finally, DNA alterations are widespread in colorectal carcinomas (Fearon & Vogelstein, 1990). Mutations can result in loss of expression of tumour suppressor genes or in dysregulation of oncogenes, and p53 (Tanaka *et al.*, 1991; Baker *et al.*, 1990) and c-*myc* (Forgue-Lafitte *et al.*, 1989) have been shown to regulate growth of colorectal carcinomas. Furthermore, an individual cell is frequently mutated at multiple sites. Growth control in such tumours would indeed be regulated by mechanisms totally independent of, and possibly refractory to, the influence of any growth factor.

This work was supported by a grant from the European Community (B MR4*-900294) to H. Lahm and a grant from the Swiss National Foundation. We thank Dr B. Sordat for critically reading the manuscript. We gratefully acknowledge the excellent technical assistance of Mrs Murielle Lorenzoni and Mrs Danuta Petral-Malec.

**References**

ANZANO, M.A., RIEMAN, D., PRICHETT, W., BOWEN-POPE, D. & GREIG, R. (1989). Growth factor production by human colon carcinoma cell lines. *Cancer Res.*, **49**, 2898.

BAKER, S.J., MARKOWITZ, S.B., FEARON, E.R., WILLSON, J.K.V. & VOGELSTEIN, B. (1990). Suppression of human colorectal carcinoma cell growth by wild-type p53. *Science*, **249**, 912.

BERDEL, W.E., DANHAUSER-RIEDL, S., STEINHAUSER, G. & WINTON, E.F. (1989). Various human hematopoietic growth factors (Interleukin-3, GM-CSF, G-CSF) stimulate clonal growth of nonhematopoietic tumor cells. *Blood*, **73**, 80.

COFFEY, R.J., GOUSTIN, A.S., MANGELSDORF SODERQUIST, A. & 4 others (1987). Transforming growth factor α and β expression in human colon cancer lines: implications for an autocrine model. *Cancer Res.*, **47**, 4590.

CULOUSCOU, J.-M., REMACLE-BONNET, M., GARROUSTE, F., MARVALDI, J. & POMMIER, G. (1987). Simultaneous production of IGF-I and EGF competing growth factors by HT-29 human colon cancer line. *Int. J. Cancer*, **40**, 646.

CZECH, M.P. (1989). Signal-transmission by the insulin-like growth factors. *Cell*, **59**, 235.

DENIZOT, F. & LANG, R. (1986). Rapid colorimetric assay for cell growth and survival. Modifications to the tetrazolium dye procedure giving improved sensitivity and reliability. *J. Immunol. Meth.*, **89**, 271.

DURRANT, L.G., WATSON, S.A., HALL, A. & MORRIS, D.L. (1991). Co-stimulation of gastrointestinal tumour cell growth by gastrin, transforming growth factor α and insulin-like growth factor-I. *Br. J. Cancer*, **63**, 67.

EL-BADRY, O.M., ROMANUS, J.A., HELMAN, L.J., COOPER, M.J., RECHLER, M.M. & ISRAEL, M.A. (1989). Autonomous growth of a human neuroblastoma cell line is mediated by insulin-like growth factor II. *J. Clin. Invest.*, **84**, 829.

FEARON, E.R. & VOGELSTEIN, B. (1990). A genetic model for colorectal tumorigenesis. *Cell*, **61**, 759.

FORGUE-LAFITTE, M.-E., COUDRAY, A.-M., BRÉANT, B. & MESTER, J. (1989). Proliferation of the human colon carcinoma cell line HT29: autocrine growth and deregulated expression of the c-*myc* oncogene. *Cancer Res.*, **49**, 6566.

HOOSEIN, N.M., KIENER, P.A., CURRY, R.C. & BRATTAIN, M.G. (1990). Evidence for autocrine growth stimulation of cultured colon tumor cells by a gastrin cholecystokinin-like peptide. *Exp. Cell Res.*, **186**, 15.

HOOSEIN, N.M., BRATTAIN, D.E., MCKNIGHT, M.K., LEVINE, A.E. & BRATTAIN, M.G. (1987). Characterization of the inhibitory effects of transforming growth factor-β on a human colon carcinoma cell line. *Cancer Res.*, **47**, 2950.

LAHM, H., FISCHER, J.R., REICHERT, Y., HEDERER, R., FALK, W., DEBATIN, K.-M. & KRAMMER, P.H. (1990). Autocrine growth factors secreted by the malignant human B-cell line BJAB are distinct from other known cytokines. *Eur. Cytokine Net.*, **1**, 41.

MACAULAY, V.M., EVERARD, M., TEALE, J.D. & 4 others (1990). Autocrine function for insulin-like growth factor I in human small cell lung cancer cell lines and fresh tumors. *Cancer Res.*, **50**, 2511.

MARKOWITZ, S.D., MOLKENTIN, K., GERBIC, C., JACKSON, J., STELLATO, T. & WILLSON, J.K.V. (1990). Growth stimulation by coexpression of transforming growth factor-α and epidermal growth factor-receptor in normal and adenomatous human colon epithelium. *J. Clin. Invest.*, **86**, 356.

MOSMANN, T. (1983). Rapid colorimetric assay for cellular growth and survival: application to proliferation and cytotoxicity assays. *J. Immunol. Meth.*, **65**, 55.

MULDER, K.M., ZHONG, Q., CHOI, H.G., HUMPHREY, L.E. & BRATTAIN, M.G. (1990). Inhibitory effects of transforming growth factor $\beta_1$ on mitogenic response, transforming growth factor α, and c-*myc* in quiescent, well-differentiated colon carcinoma cells. *Cancer Res.*, **50**, 7581.

MULDER, K.M., RAMEY, M.K., HOOSEIN, N.M. & 4 others (1988). Characterization of transforming growth factor-β-resistant subclones isolated from a transforming growth factor-β-sensitive human colon carcinoma cell line. *Cancer Res.*, **48**, 7120.

OHMURA, E., OKADA, M., ONODA, N., KAMIYA, Y., MURAKAMI, T. & SHIZUME, K. (1990). Insulin-like growth factor I and transforming growth factor α as autocrine growth factors in human pancreatic cancer cell growth. *Cancer Res.*, **50**, 103.

PIGNATELLI, M. & BODMER, W.F. (1989). Integrin-receptor-mediated differentiation and growth inhibition are enhanced by transforming growth factor-β in colorectal tumour cells grown in collagen gel. *Int. J. Cancer*, **44**, 518.

RODECK, U., HERLYN, M., HERLYN, D. & 5 others (1987). Tumor growth modulation by a monoclonal antibody to the epidermal growth factor receptor: immunologically mediated and effector-cell independent effects. *Cancer Res.*, **47**, 3692.

SCALA, G., MORRONE, G., TAMBURRINI, M. & 4 others (1987). Autocrine growth function of human interleukin 1 molecules on ROHA-9, an EBV-transformed human B cell line. *J. Immunol.*, **138**, 2527.

SHIPLEY, G.D., CHILDS, C.B., VOLKENANT, M.E. & MOSES, H.L. (1984). Differential effects of epidermal growth factor, transforming growth factor, and insulin on DNA and protein synthesis and morphology in serum-free cultures of AKR-2B cells. *Cancer Res.*, **44**, 710.

SPORN, M.B. & TODARO, G.J. (1980). Autocrine secretion and malignant transformation of cells. *New Engl. J. Med.*, **303**, 878.

SUARDET, L., LAURENT, P., TEVAERAI, H., GIVEL, J.-C. & ODARTCHENKO, N. (1990). Effect of granulocyte-macrophage colony-stimulating factor on *in vitro* growth of human colorectal cancer cells. *Exp. Hematol.*, **18**, A250.

SUARDET, L., GROSS, N., GAIDE, A.-C., BECK, E. & ELIASON, J.F. (1989). Epidermal growth factor responsiveness of a new human neuroblastoma cell line. *Int. J. Cancer*, **44**, 661.

TANAKA, T., OSHIMURA, M., KIKUCHI, R., SEKI, M., HAYASHI, T. & MIYAKI, M. (1991). Suppression of tumorigenicity in human colon carcinoma cells by introduction of normal chromosome 5 or 18. *Nature*, **349**, 340.

TRICOLI, J.V., RALL, L.B., KARAKOUSIS, C.P. & 4 others (1986). Enhanced levels of insulin-like growth factor messenger RNA in human colon carcinomas and liposarcomas. *Cancer Res.*, **46**, 6169.

TSAI, S.-C.J. & GAFFNEY, E.V. (1987). Modulation of cell proliferation by human recombinant interleukin-1 and immune interferon. *J. Natl. Cancer Inst.*, **79**, 77.

TWENTYMAN, P.R. & LUSCOMBE, M. (1987). A study of some variables in a tetrazolium dye (MTT) based assay for cell growth and chemosensitivity. *Br. J. Cancer*, **56**, 279.

WAKELING, A.E., NEWBOULT, E. & PETERS, S.W. (1989). Effect of antioestrogens on the proliferation of MCF-7 human breast cancer cells. *J. Mol. Endocrinol.*, **2**, 225.

WONG, S.T., WINCHELL, L.F., MCCUNE, B.K. & 5 others (1989). The TGF-α precursor expressed on the cell surface binds to the EGF receptor on adjacent cells, leading to signal transduction. *Cell*, **56**, 495.